# How to Model Rheumatoid Arthritis in Animals: From Rodents to Non-Human Primates

**DOI:** 10.3389/fimmu.2022.887460

**Published:** 2022-05-25

**Authors:** Ting Zhao, Zhaohu Xie, Yujiang Xi, Li Liu, Zhaofu Li, Dongdong Qin

**Affiliations:** ^1^ School of Basic Medical Sciences, Yunnan University of Chinese Medicine, Kunming, China; ^2^ Ge Jiu People’s Hospital, Yunnan Honghe Prefecture Central Hospital, Gejiu, China

**Keywords:** rheumatoid arthritis, animal models, rodents, NHPs, autoimmune diseases

## Abstract

Rheumatoid arthritis (RA) is a chronic inflammatory autoimmune disease influenced by both genetic and environmental factors. At present, rodent models are primarily used to study the pathogenesis and treatment of RA. However, the genetic divergences between rodents and humans determine differences in the development of RA, which makes it necessary to explore the establishment of new models. Compared to rodents, non-human primates (NHPs) are much more closely related to humans in terms of the immune system, metabolic conditions, and genetic make-up. NHPs model provides a powerful tool to study the development of RA and potential complications, as well as preclinical studies in drug development. This review provides a brief overview of the RA animal models, emphasizes the replication methods, pros and cons, as well as evaluates the validity of the rodent and NHPs models.

## Introduction

Rheumatoid arthritis (RA) is a chronic inflammatory disease characterized by joint swelling, joint tenderness, and destruction of synovial joints, leading to severe disability. Worldwide, the prevalence of RA ranges from 0.5% to 1% (1–3). Despite advances in our understanding of RA pathogenesis and improvements in RA treatment, some RA patients remain refractory to disease-modifying antirheumatic drugs (4). Therefore, we urgently need to deeply explore the pathogenesis of RA and further search for new therapeutic strategies. Currently, rodents are usually used to study the progression and pathogenesis of RA (5). However, the genetic divergences between rodents and humans determine differences in the development of RA. There is a growing awareness that the evolutionary gap between inbred rodents and outbred humans is too wide for direct translation from rodents to humans.

Crucially, differences in metabolic pathways between rodents and humans further hamper the direct translation of new therapeutic strategies into the clinic. Some biotherapeutic drugs are ineffective in rodent models. In addition, any beneficial effects proven in rodent models do not guarantee the same or similar therapeutic effects in RA patients. Therefore, RA can be modeled using another specie more closely related to humans, such as non-human primates (NHPs) (6). Recently, significant progress has been made in the study of using NHPs to establish RA models.

This mini review provides a brief overview of the RA animal models, emphasizes the replication methods, pros and cons, as well as evaluates the validity of the rodent and NHPs models.

## Rodent Animal Models Used in the Study of RA

### Induced Arthritis Models

A variety of rodent models have been established to study the etiology and pathogenesis of RA. Rodent RA models mainly include collagen-induced arthritis (CIA), adjuvant arthritis (AA), delayed-type hypersensitivity arthritis (DTHA), anti-citrullinated peptides antibodies (ACPA)-mediated arthritis, etc.

#### Collagen-Induced Arthritis

The CIA model is one of the most widely used rodent models of RA, mainly characterized by the destruction of self-collagen tolerance and the production of autoantibodies (7). In 1980, Courtenay et al. proposed using type II collagen (CII) to induce a mouse model of arthritis (8). In mice, bovine CII and complete Freund’s adjuvant (CFA) were emulsified and injected into the base of the tail to replicate human RA. Rats were modeled by intradermal injection of CII and incomplete Freund’s adjuvant (IFA) at multiple sites, as well as boosted by intraperitoneal injection one week later (9). The CIA model has become an essential element for testing and developing new biological-based therapies (10, 11). It was once recognized as the best model for RA, but only under certain experimental conditions can it be modeled successfully. Furthermore, the CIA model cannot show RA fluctuations and recurrences, vasculitis symptoms, subcutaneous nodules, or serositis.

#### Adjuvant-Induced Arthritis

AA is an earlier classical RA animal model, which is widely used in the study of the pathogenesis of RA and the evaluation of anti-arthritis drugs. The bacteriologist Freund created the adjuvant-induced arthritis model in the 1950s, also called Freund’s adjuvant arthritis (12). The CFA-induced arthritis model was established through unilateral subcutaneous injections of 0.1 mL CFA into the hindfoot or tail root (13). It is suitable for studying molecular mechanisms between T cells and subpopulations (14). However, this model lacks the chronic progressive nature of RA, and the pathological changes are self-limiting.

#### Other Induced models

The DTHA model is a pharmacologically relevant monoarthritis model. It is highly reproducible and has a high incidence rate (15). However, DTHA is self-limiting and inconsistent with the chronic progressive characteristics of RA. A study has shown that a citrullinated fibrinogen-specific T cell line enhances autoimmune arthritis in a mouse model of RA (16). The ACPA-induced arthritis murine model is significant for the in-depth study of citrullinated protein antibody-positive RA pathogenesis and the development of new drugs (17–19). The CAIA model is induced by administering a cocktail of endotoxin and monoclonal antibodies, which is widely used to study the pathogenesis of RA and evaluate therapeutic effects (20). A pristane-induced arthritis model is an essential tool for understanding the mechanism of inflammatory joint disease, especially those depending on T-cell signaling pathways (21, 22). Moreover, antigen-induced arthritis (23), proteoglycan-induced arthritis (24, 25), streptococcal cell wall-induced arthritis (26), glucose-6-phosphate isomerase-induced arthritis (27), and other RA animal models have all contributed to the research of RA to a certain extent (28, 29), see **Table 1** for more details.

**Table 1:** Animal models of RA.

Species	Methods	Induced/spontaneous	Animals	Disease cycle	Applications	Validity	References
Rodents	CIA, immunization with CII	Induced	Rats, mice	14-60 days	New drug treatment and treatment target detection	Face, construct and predictive validity	(7–11)
	AA, immunization with adjuvant	Induced	Rats	14-45 days	Molecular mechanism research between T cells and subpopulations	Face and construct validity	(12–14)
	DTHA, immunization with methylated bovine serum albumin, with the modification that a cocktail of type II collagen monoclonal antibodies	Induced	Mice	24-48 h	Preclinical screening of novel drugs targeting RA	Face and predictive validity	(15)
	ACPA-mediated arthritis, immunization with citrullinated peptides	Induced	Mice	3-5 weeks	Study the model of ACPA mediated arthritis	Face and construct validity	(16–19)
	CAIA, systemic administration of mixtures of antibodies	Induced	Mice	3–8 days	Suitable for strains or genotypes that are not suitable for CIA	Face and construct validity	(20)
	PIA, single intradermal injection of pristane in rats; two injections of pristane at an interval of 50 days in mice	Induced	Rats, mice	14-180 days	Verify the efficacy of new anti-arthritis drugs	Face, construct and predictive validity	(21, 22)
	AIA, immunization with antigen	Induced	Rats, mice	1-3 days	Study humoral and cellular immune responses	Face and construct validity	(23)
	PGIA, immunization with proteoglycan (PG)	Induced	Rats, mice	21-28 days	Study new drugs for arthritis	Face, construct and predictive validity	(24, 25)
	SCWA, intra-articular injection of SCW fragments	Induced	Rats, mice	0-30 days	Study the acute or flare reaction in arthritis	Face and construct validity	(26)
	G6PI, injection of G6PI	Induced	Mice	9-72 days	Study the mechanism of G6PI in the pathogenesis of RA	Face and construct validity	(27)
	COMP, subcutaneous injection of COMP and ICFA emulsifier in tail	Induced	Rats, mice	13-43 days	Suitable and alternative models for the pathogenesis of arthritis	Face and construct validity	(28, 29)
	K/B×N arthritis, crossing of Aβ^g7^ Transgenic BABL/c with the same strain B6.H2^g7^	Spontaneous	Mice	25-35 days	Study the mechanism of IFN treatment for RA	Face and construct validity	(30, 31)
	SKG arthritis, ZAP-70 gene locus mutation	Spontaneous	Mice	1-8 weeks	Assess the effect of drugs on RA bone changes	Face and predictive validity	(32)
	IL-1ra-/- arthritis, inject IL-1ra expression vector into mouse fertilized eggs	Spontaneous	BALB/c mice	5-8 weeks	Study the role of cytokines in the pathogenesis of RA	Face and construct validity	(33)
	TNF-α transgenic, inject the fragment containing the 3'-modified human TNF gene into the fertilized egg of mice	Spontaneous	Mice	3-4 weeks	Study the role of related cytokines in TNF arthritis, such as nuclear factor κB receptor activating factor ligand	Face and construct validity	(34)
Non-human primates	CIA, immunization with CII	Induced	Macaques, marmosets	14-126 days	Test new human specific therapeutics	Face, construct and predictive validity	(4, 6, 35–41)
	ACPA-mediated arthritis, immunization with citrullinated peptides	Induced	Macaques	3-34 weeks	Study the model of ACPA mediated arthritis	Face and construct validity	(42, 43)
	Spontaneous RA model	Spontaneous	Macaques	5 years old	Mechanism and translational research	Face and construct validity	(44, 45)

CIA, collagen-induced arthritis; AA, adjuvant-induced arthritis; COMP, cartilage oligomeric matrix protein-induced arthritis; PIA, pristane induced arthritis; AIA, antigen-induced arthritis; PGIA, proteoglycan-induced arthritis; CAIA, collagen antibody induced arthritis; G6PI, glucose-6-phosphate isomerase-induced arthritis; SCWA, streptococcal cell wall-induced arthritis; ACPA, anti-citrullinated peptides antibodies.

### Spontaneous Transgenic Model

In addition to induced arthritis models, the spontaneous RA can also be modeled using transgenic rodents. K/BxN mice have a rapid onset of disease in a short period, which is crucial for the rapid screening of anti-arthritis drugs and new targets (30, 31). The SKG mouse transgenic model belongs to T cell-mediated chronic autoimmune polyarthritis with slow progression (32). IL-1ra gene deficiency causes autoimmunity and joint-specific inflammation. IL-1ra -/- transgenic mice often present clinical features such as inflammatory infiltration and pannus formation (33). The phenotype of the TNF-α transgenic model is stable, and the progression of the disease presents chronic inflammation, which is conducive to the development of drugs and the improvement of diagnosis and treatment technology (34). Most of these models lack or are genetically modified with one specific gene. Considering RA’s polygenic traits, one targeted gene makes the simulation of spontaneous disease models narrower and simpler. These models are used to study the role of a specific gene in RA. The models are hereditary and can be continuously expressed in offspring. Therefore, they are used as tools to study the therapeutic effects for mice that are prone to develop joint inflammation spontaneously.

## NHPs Model for RA

### Induced Arthritis Models

There are currently few NHPs models to study RA’s etiology and pathogenesis and guide the evaluation of RA therapeutic drugs. NHPs RA models mainly include CIA and citrullinated peptide-induced arthritis (**Table 1**).

#### Collagen-Induced Arthritis

CIA can be elicited in susceptible strains of NHPs by immunization with CII, making it a valuable model to better assess the efficacy of novel therapeutic targets and aid their transition through the primary stages of preclinical development (4). An emulsion containing CII and complete Freund’s adjuvant was injected into 10 to 20 sites on the base and back of each macaque’s tail. Three weeks later, macaques were again injected with CII in incomplete Freund’s adjuvant. CII can induce autoimmune polyarthritis with certain RA clinical and immunological features in macaques and marmosets. Like the rodent models, the CIA induction in macaques depends on the synergy of delayed hypersensitivity and immune complex-mediated inflammation (35). Both macaque and marmoset RA models have found that collagen-specific antibodies, especially IgM isotype collagen-specific antibodies, play an essential role in developing CIA models. In about 60% of the monkeys, CIA could be induced, whereas the remaining 40% appeared completely asymptomatic, even after repetitive booster immunizations with CII in incomplete Freund’s adjuvant (6, 36). A study found that rhesus monkeys immunized with collagen exhibit varying degrees of RA symptoms, including joint swelling and stiffness, increased proliferation of anti-type II collagen antibodies, and damage to articular cartilage tissues, consistent with observation in the murine system (37). At the same time, mononuclear granulocytes and C-reactive protein (CRP) increased significantly and remained at a high level. The X-ray images of CIA rhesus monkeys are similar to the results of clinical RA. They both show swelling of soft tissue around the affected joints, localized osteoporosis, joint space narrowing, and bone erosion (38). Compared with rhesus monkeys, the bones and cartilage of common marmosets are less damaged, showing unique extra-articular manifestations of inflammation at the periosteum and subcutaneous tissues (39). The common marmosets are closer to the chronic RA with respect to the chronic disease course and pathomorphological presentation than the more acute monophasic and destructive CIA model in macaques (40, 41). It can serve as a suitable model to bridge the gap between rodents and primates.

#### Citrullinated Peptides-Induced Arthritis

In the late 1990s, researchers discovered a new autoantibody ACPA in serum samples of RA patients, which has reasonable clinical diagnostic specificity for RA. ACPA antibodies are present in 75% of RA patients, and the specificity of diagnosis is as high as 98% (42). The target antigen of ACPA is a type of citrullinated antigen produced by post-translational modification of protein (protein citrullination). Usually, protein citrullination is catalyzed by protein arginine deiminase (46). ACPA stimulates mononuclear macrophages to produce TNF-α and other pro-inflammatory cytokines to promote inflammatory progress. Studies have found that a short peptide of cyclic citrullinated vimentin conjugated to hemocyanin induces arthritis, which symptoms more similar to the development of human RA in rhesus monkeys (43). The emulsion of cyclic citrullinated vimentin conjugated to hemocyanin was injected subcutaneously into the back of macaques (10 sites, a total of 2 mg) and the macaques received the same dose of booster injection on the fourth and eighth weeks after the first immunization. The prominent presentation was slowly developing joint deformation until the functional impairment of typical joint deformity appeared in the later period of experimental observation, which is similar to the clinical joint swelling and pain of some RA patients. Consistently, the joint deformation is not apparent and is significant in the later stage of the disease. A recent study developed an ACPA-mediated arthritis macaque model based on immunization against citrullinated peptides (42). Macaques can display the symptoms of chronic persistent joint synovitis and persistent bone destruction, reflecting the chronic erosion effect of human RA.

### Spontaneous Model

Spontaneous arthritis has been found in NHPs models, mainly macaques (approximately 20%). The IL-7 and IL-15 genes of rhesus macaques have high similarity (96%) and functional homology with humans. There is three MHC class I loci and 3 MHC class II loci (HLA DP, DQ, and DR) in human beings, and macaques have 2 MHC-I loci and 3 MHC class II loci (DP, DQ, DR). Unlike humans, macaques may have several MHC-I alleles on each chromosome and may have a significant cross, which is expected to increase haplotype diversity (47). It was previously shown that cynomolgus monkeys from China (5 years old) can spontaneously develop polyarthritis, local inflammation, significant joint swelling, elevated neutrophils, monocytes, and serum CRP (44). Recent studies have revealed that the heart function of rhesus monkeys with spontaneous RA showed progressive deterioration, and the receptor-interacting protein kinase 1 that binds to the voltage-dependent anion-selective channel 1 is upregulated (45). The innate NHPs animal model of RA can simulate the entire development process of RA from early stage to late stage without external intervention, avoiding artificial injury. Moreover, the pathogenesis and pathological injury are very similar to primary human RA, which can meet the needs of RA at different stages. This model is suitable for studying the effect of specific treatments on the whole course of RA and determining the long-term effects of certain medicines. Some studies have sequenced the macaques’ genome and obtained transgenic macaques (48). We speculate that the genetic engineering model of NHPs may become a new direction of medical research in the future and provide vital information for RA research.

It is worth noting that NHPs should be used following the Four Rs tenet, including replacement, reduction, refinement and rehabilitation. Replacement refers to methods which avoid or replace the use of NHPs with lower animals such as rodents. Reduction means reducing the number of NHPs to a minimum to obtain information from fewer numbers. Refinement is the way experiments are carried out in order to make sure NHPs suffer as little as possible (e.g., improvement in experimental conditions, anesthesia and analgesia for pain relief). Rehabilitation refers to necessary after-care and/or rehabilitation of NHPs post-experimentation. It is also strongly recommended to use humane endpoints to prevent, alleviate, or reduce pain and distress of NHPs. When experiments are completed, animals with untreatable conditions should not experience undue pain or distress and are euthanized in a timely fashion.

## Assessing Validity in Animal Models of RA

Developing effective animal models for complex autoimmune diseases, including RA, has proved very challenging, and the various symptoms of RA are challenging to model in any non-human species. There are many possible approaches to create RA animal models, and each animal model should be evaluated based on face, construct, and predictive validity to determine their potential values as a valid model for RA. Face validity is meant by similarities between the model’s outcome measures and the phenotypes of the human RA. The construct validity is understood to be the correlation between the method of modeling and the etiology of human RA. The predictive validity refers to the model’s response to a therapeutic drug used to treat human RA disease (49).

### Face Validity

An animal model with high face validity will produce symptoms similar to human diseases. Animal models are mainly determined by general morphological observation, imaging examination, pain threshold, arthritis score index, and laboratory biomarkers like human RA diagnosis. The primary goal of a valid animal model of RA is to exhibit pain, inflammation, joint destruction, and various antibodies.

#### Pain

Persistent joint pain and tenderness are the main clinical manifestations of RA. Evaluation of potential analgesic therapeutics and elaboration of the neurobiology of pain have heavily relied on pain models developed in rodents. In rodents, hyperalgesia is typically manifested by decreased mechanical withdrawal threshold, thermal withdrawal latency, and expression of pain-related factors (50). In addition, methods to induce chronic pain-like states and quantify changes in nociception that have been developed in rodents could be adapted to NHPs (51). NHPs make better responses to pain than rodents through various facial expressions, vocalizations, and body positions, just like in humans. This suggests that NHPs have higher face validity compared to rodents.

#### Inflammation

The serum level of CRP is a handy marker for systemic inflammation. Serum CRP concentration can directly reflect the intensity of the RA pathological process and can be used as an early marker for disease onset (52). The serum level of CRP is closely related to IL-6. It can induce the production of autoantibodies, and its consistency with the treatment response is more significant than the erythrocyte sedimentation rate (53). In a rhesus monkey CIA model, serum IL-6 levels were also related to clinical and laboratory parameters such as CRP, MMP3, anti-CII antibodies, STS score, and creatinine (37). In addition, interferon γ is a potent pro-inflammatory factor, which is widely involved in inflammatory reactions and may play an essential role in the pathogenesis of RA (54).

#### Joint destruction

Urinary excretion rates of hydroxylysylpyridinoline (HP) and lysylpyridinoline (LP) can serve as biomarkers for joint destruction. About 95% of the cross-links in the joint cartilage of the rhesus monkey consist of HP, while the HP/LP ratio in bone is 3.8% (55). The excretion rate of HP and LP can increase during the active period of the CIA. Serum alkaline phosphatase (ALP) is an effective index of bone metabolism, which is mainly produced in the liver and osteoblasts and is related to RA (56). Changes in ALP may indicate increased bone metabolism. MMP-3 is one of the most significant degradation enzymes of the cartilage matrix. It can be used as a laboratory indicator to evaluate joint damage status and treatment effect, proving superior to other traditional and conventional laboratory indicators (57).

#### Antibodies

RA markers with clinical significance include various autoantibodies, rheumatoid factors, and ACPA. Among them, ACPA plays a vital role in diagnosing and treating RA. Commonly used citrullinated protein-related antibodies include anti-cyclic citrullinated peptide (anti-CCP), anti-keratin (aka), and anti-perinuclear factor (APF) (58). Erythrocyte sedimentation rate and CRP are also associated with RA. Combined with different markers, the comprehensive diagnosis of RA has more clinical significance, which is helpful to improve the sensitivity and accuracy of RA animal model diagnosis.

### Construct Validity

Construct validity is very important in assessing the validity of animal models of RA. Early efforts to develop animal models of RA were hampered by a lack of understanding of the underlying causes of RA. Animal model experiments are now permitted to assess risk factors (environmental factors, genetic susceptibility, and immune system disorders) identified through RA patient studies. It is worth noting that RA rodent models are all established under certain experimental conditions, focusing on one or several factors, and cannot fully reflect all the characteristics of human RA. However, the macaques can spontaneously develop RA with the increase of age (45). Therefore, compared with rodents, NHPs may demonstrate better construct validity.

### Predictive Validity

Predictive validity refers to treatments that alleviate human disease symptoms and reverse pathological features in animal models. With the development of new drugs for RA, the predictive validity of animal models for RA may specifically target pivotal cytokines in the inflammatory cascade in RA and its complications, such as dyslipidemia and anemia (59). Although rodent models developed over the past few decades have helped to identify new therapeutic targets for RA, NHPs are more closely related to humans in genetic evolution and immunological responses, and therefore represent a model with good predictive validity for evaluating the safety and efficacy of new therapies.

In conclusion, evaluating the validity of RA animal models will help us further understand RA’s pathogenesis and develop safe and effective therapeutic strategies. However, an ideal animal model of RA must balance biological feasibility and practical considerations. NHPs are often expensive and difficult to reproduce. In rodent models, up to 10 mice per analysis are used to see significant treatment effects, whereas the minimum number of NHPs is around 3. Despite some limitations, NHPs have substantial advantages in the face, construct, and predictive validity, which are more suited to pre-clinical drug development studies for RA.

## Conclusion and Perspectives

The cornerstone of RA research is the development of animal models to mimic human autoimmune system disorder and test novel pharmacologic targets. Preclinical and clinical studies are required to develop and demonstrate the efficacy of new drugs for RA patients. As far as animal model research is concerned, there is no animal model with all the characteristics of human RA. Each model has its own features.

Statistics indicated that approximately 90% of the animals applied in the research were mice, rats, and other rodents (60). However, the genetic divergences between rodents and humans determine differences in the development of RA. More than 70 million years ago, rodents and humans diverged from each other (61). Although RA can be modeled in rodents, their immune response may differ from NHPs or humans. The joint structure of rodents is too small, which is not conducive to implementing various imaging methods. A unique feature of NHPs is the possibility of obtaining complete clinical chemistry and hematological analysis, giving additional insights into the disease status and the general physical condition of the animal (62). Compared with rodents, NHPs biology is most phenotypically comparable to humans biology, including genetics, immunities, metabolomics, and pathologies (63). For example, the expression of toll-like receptors (TLRs) among different dendritic cells (DC) subsets is identical between humans and macaques but differs from murine DCs (64). In 2007, the cynomolgus macaque genome was sequenced, and it is 93% similar to the human genome, making them valuable animal models compared to rodents for the study of human diseases, e.g., RA (65). It can better simulate the common parts of human RA that produce symptoms. Moreover, the characterization of RA models in NHPs will provide insights into mechanisms and new therapies that cannot be addressed by other animal models.

Therefore, NHPs may be an ideal natural model for clinical RA, or they will become an excellent resource for studying the pathogenesis of RA and guiding the search for new therapeutic targets.

## Author Contributions

LL, ZL and DQ raised the idea and supervised the work. TZ, ZX and YX performed the literature search and data analysis. TZ, LL, ZL and DQ wrote the manuscript. LL, ZL and DQ revised the manuscript. All authors contributed to the article and approved the submitted version.

## Funding

National Natural Science Foundation of China (31960178, 81960863, 81960870, 82160901); Construction Project of National Traditional Chinese Medicine Clinical Research Base (2018 No. 131); Yunnan Provincial Fund for Medical Research Center: Clinical Evaluation and Basic Research on the Treatment of rheumatoid arthritis and gout by Traditional Chinese medicine (202102AA310006); Clinical Trial for the Treatment of Rheumatoid Arthritis with Warming yang and Smoothening Meridians (201507001-07, registration number: ChiCTR-INR-16010290); Clinical Cooperative Project of Chinese and Western Medicine for Major and Knotty Diseases; Yunnan Provincial Key Laboratory Construction Project Funding; Yunnan Provincial Key Laboratory of Chinese Medicine Rheumatology and Immunology; Yunnan Provincial “Ten Thousands Program” Famous Doctor Special; Yunnan Province Qingguo Wang Expert Workstation Construction Project (202005AF150017); Yunnan Applied Basic Research Projects-Union Foundation (2019FF002(-031)); Applied Basic Research Programs of Science and Technology Commission Foundation of Yunnan Province (2019FA007); Scientific Research Fund Project of Yunnan Provincial Department of Education (2021Y461).

## Conflict of Interest

The authors declare that the research was conducted in the absence of any commercial or financial relationships that could be construed as a potential conflict of interest.

## Publisher’s Note

All claims expressed in this article are solely those of the authors and do not necessarily represent those of their affiliated organizations, or those of the publisher, the editors and the reviewers. Any product that may be evaluated in this article, or claim that may be made by its manufacturer, is not guaranteed or endorsed by the publisher.
